# Clinical usability study of a home-based self-administration transcranial direct current stimulation for primary dysmenorrhea: A randomized controlled trial

**DOI:** 10.1371/journal.pone.0301851

**Published:** 2024-05-02

**Authors:** Yvinna T. Rodrigues, Tatiana C. L. A. Silva, Emilė Radytė, Ervinas Bernatavičius, Alexander A. Cook, Maria L. A. S. Carvalho, Luiza E. S. Macedo, Joyce M. P. Oliveira, Talita D. Martins, Maria E. Fonseca, Maria T. A. B. C. Micussi, Rodrigo Pegado

**Affiliations:** 1 Graduate Program in Physical Therapy, Federal University of Rio Grande do Norte, Natal, Brazil; 2 Samphire Neuroscience Ltd, London, United Kingdom; 3 Department of Physical Therapy, Federal University of Rio Grande do Norte, Natal, Brazil; 4 Graduate Program in Health Science, Federal University of Rio Grande do Norte, Natal, Brazil; University of Bologna: Universita di Bologna, ITALY

## Abstract

This study tested the usability of a home-based self-administration transcranial direct current stimulation (tDCS) device designed specifically for women’s health needs. This is a single center triple blinded clinical usability study for a new wireless, Bluetooth-controlled wearable tDCS device for women’s health. The study aims to evaluate the usability and effective blinding of a home-based tDCS system. A total of forty-nine women of reproductive age were randomly allocated (1:1) to receive one session of active tDCS (n = 24) or sham tDCS (n = 25) over the motor and dorsolateral prefrontal cortex. Each participant self-administered one 20-minute session without supervision following guidance on a software application alone. The System Usability Scale (SUS) and the Patient Global Impression of Change (PGIC) were used to evaluate the usability of the system. Regardless of sham or active conditions, all users found the system easy to use without the support of researchers. Usability scores were considered to be “excellent” in both groups and no significant difference was found between sham and active groups showing effective blinding of the device (Active group: 93.7 (83.1–97.5); Sham group 90 (86.2–95) p = 0.79) and PGIC (Active group: 2 (1–2.75); Sham group 2 (1–2) p = 0.99) using an unpaired t-test or non-parametric statistical tests accordingly. The new Bluetooth-controlled wearable tDCS device is easy, safe to use and completely controlled by a smartphone app. This device is focused on women’s health and will be tested as an alternative treatment for chronic pelvic pain and mood disturbance associated with menstrual cycles in further research.

## Introduction

Dysmenorrhea is the medical term used to describe chronic recurrent pelvic pain commonly known as menstrual cramps. It can be classified as primary (PD) or secondary dysmenorrhea [1]. PD is one of the most prevalent gynecologic conditions and is characterized by the main symptom of pain in the previous period and during menstrual bleeding in the absence of any other identifiable cause [1]. In addition to pain, PD can present with symptoms such as anxiety, depression, affective disorders, nausea, migraine and episodes of diarrhea [2]. About 17 to 90% of women will experience some episode of pain during their reproductive cycle [2]. Some women report a meaningful degree of disability during the initial period of their menstrual cycle, which reflects negatively on school, work activities and on their quality of life [1,2].

Long-term PD is associated with changes in brain metabolism and functional connectivity in areas related to pain modulation [3–6]. Such regions as the primary motor cortex (M1), medial prefrontal cortex (mPFC), posterior cingulate cortex and insula show an abnormal functional pattern [3]. These areas are responsible for emotional affective control of pain, cognitive control and pain generalization [3]. In addition, women with PD have increased theta activity (4–7 Hz) in pain-related brain areas [4]. Painful experience is also related to depression and anxiety, a fact that may be a fundamental factor in understanding the role of theta activity in the sensory and emotional processing of pain in PD [4].

Non-invasive brain stimulation (NIBS) has been used in several clinical conditions aiming to improve motor, cognitive, affective-behavioral and physical function [7]. The clinical application of NIBS has been gaining prominence in new consensuses and guidelines in physical and cognitive rehabilitation aimed to improve or restore function in chronic diseases [7–9]. Among the various non-invasive brain stimulation techniques, transcranial direct current stimulation (tDCS) emerges as a potentially scalable solution. Through a constant microcurrent of intensity between 1 to 4 mA applied to the scalp, changes in cortical excitability occur according to stimulation parameters related to exposure time, intensity and electrode assembly, leading to observed clinical effects [7,10].

Remote rehabilitation processes are the frontier of care among healthcare professionals and patients, including tDCS treatment. Remote tDCS solutions were widespread and advocated during the Covid-19 pandemic period of social isolation and appear to be following a clinical trend [11]. Requiring participants to attend research sites, hospital facilities or rehabilitation clinics for multiple treatment sessions (which can be as many as 20 or more), however, is a logistically challenging process. For many patients, it can mean high transportation costs, travel time, food costs and absenteeism for work or school, and therefore overall reduced access to treatment. A remote tDCS treatment model, with low adverse effects, that is safe and easy to use, could make the technique easier to adhere and apply, as well as provide another treatment option for the many women living with PD. To date, several remote supervision tDCS protocols have been performed, focusing on chronic pain, musculoskeletal and behavioral disorders [12–14]. However, no device ever focused on women’s health needs, including patients with PD. A home-based self-administration tDCS was developed as a user-friendly, novel home-based tDCS system focused on addressing PD symptoms based on established stimulation parameters and electrode montages. The home-based self-administration tDCS utilized in this study is a wireless, Bluetooth-controlled wearable device designed to resemble a women’s hairband and controlled via an accompanying app. The tDCS device provides the user with information about the session, verifies for any contra-indications, sets up appropriate session parameters, starts, pauses and stops the session, helps with troubleshooting, and collects user feedback following a successfully completed session.

This is a preliminary clinical usability and feasibility-only study. The study aimed to evaluate the usability of the home-based self-administration of tDCS system with a focus on women’s health. The specific aims of this study were to: evaluate the score of System Usability Scale (SUS) and Patient Global Impression of Change; after the use of a new home-based self-administration tDCS system.

## Materials and methods

### Design

This was a single center double blinded clinical usability study at Federal University of Rio Grande do Norte (UFRN), Natal, Brazil. The study was previously approved by the Research Ethics Committee of the UFRN under number 5.508.364 and registered with the Brazilian Clinical Trials Registry (RBR-56w7h2p). Participants signed an informed consent form before participation. Data collection about usability study was carried out in October 2022. This study specifically utilized data from a specific component of a longer clinical trial. While the larger trial encompasses various aspects and objectives, the focus of the first investigation is solely on the usability study of a home-based tDCS system.

### Recruitment and eligibility criteria

The recruitment for the study was carried out through electronic media (Facebook^™^ and Instagram^™^) and posters placed around the university. Participants were recruited upon spontaneous demand. Inclusion criteria admitted participants, who: (1) historic of PD; (2) were aged from 18 to 45 years, (3) had a regular menstrual cycle from 28 to 32 days, (4) were not lactating, (5) had no history of brain surgery, tumor, or intracranial metal implantation, and (6) had no history of chronic genitourinary infections, alcohol, or drug abuse. Exclusion criteria were: (1) patients presenting with a history of dizziness or epileptic disease, (2) pregnancy, (3) metal implants in the head.

The study protocol specifically targeted the late follicular or luteal phase of the menstrual cycle, ensuring that participants were free from pain or mood disturbances during the study procedures. This is attributed to the focus of the study, which is centered on clinical usability and feasibility rather than efficacy assessment.

### Randomization and blinding

**Fig 1:**
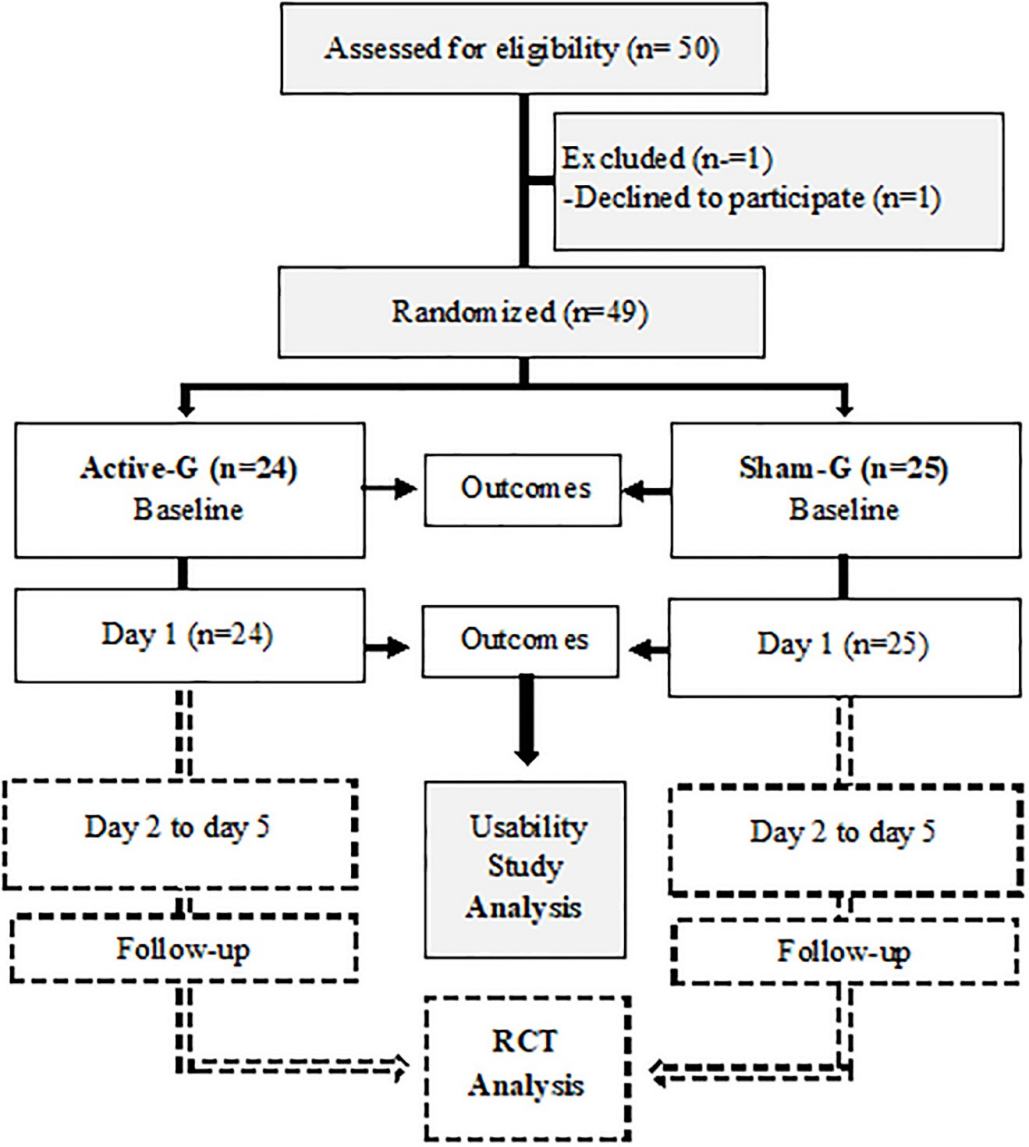
Flowchart of the study. RTC: Randomized clinical trial. The dashed lines correspond to phases that are still ongoing.

A total of fifty women participated in the study and one declined to participate. Forty-nine participants were enrolled by the blinded investigators and randomly allocated (1:1) to receive active tDCS (n = 24) or sham tDCS (n = 25) (Fig 1). According to Macefield, this group size is a sensible number to consider for usability studies [15]. Regarding usability studies, Macefield recommended that a sample of 50 users could yield a minimum of 98% problem detection. Stratified randomization was done with a block size of 10 participants based on their order of entry into the study. An appropriate software tool was employed to randomly assign each participant to either the active or sham group. An external blinded research assistant generated the allocation sequence. Participants and evaluators were blinded to group allocation throughout the trial.

### Intervention

**Fig 2:**
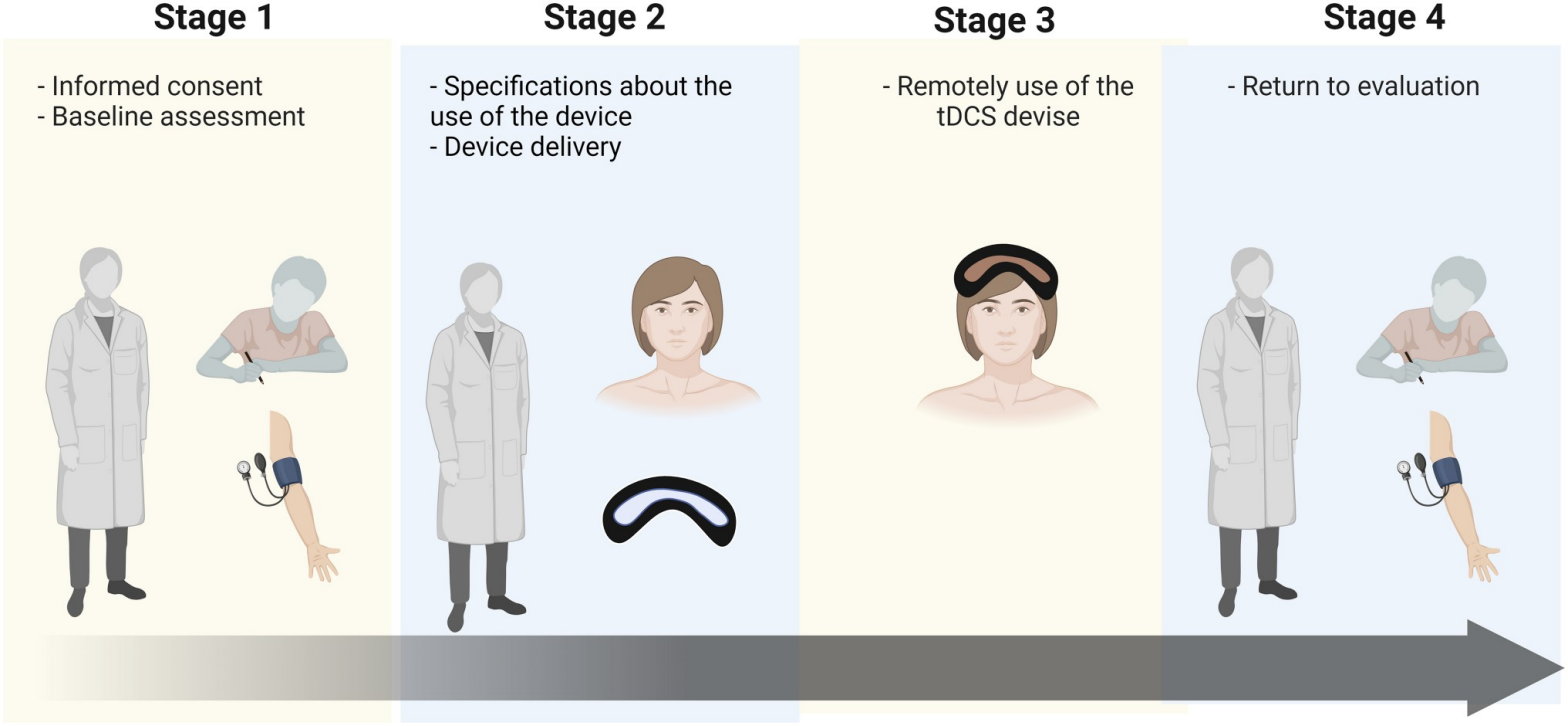
Timeline of the clinical usability study protocol.

The clinical usability study consisted of a single-day day assessment and intervention (for each participant) with 4 stages: (1) initial meeting with the participants to introduce the project, complete informed consent procedures and baseline evaluation, (2) exposure and specifications about the use of the device, (3) use of the device by the participant without the supervision of the researchers, (4) usability evaluation post-use (Fig 2). The baseline assessment was performed by a blinded evaluator, individually and in a temperature-controlled and noise-free room. All participants answered the questionnaires with sociodemographic and clinical information aimed to characterize the sample (pain, depression, anxiety, positive and negative affect, premenstrual symptoms and quality of life).

After the baseline assessment, the participants were instructed on how to use the tDCS device. The Samphire Neuroscience tDCS designed for scientific research was used. All participants received training with the research team (physiotherapists) in order to learn how to handle the device, how to place it on the head, how to control it using the Samphire App for smartphone, as well as best practices for the preservation and conservation of the device (such as leaving the tDCS in a safe place, not over-exposing it to the sun or a heat source), and risks and guidelines related to expected adverse effects of use (redness at the site, itching where the electrodes are in contact with the skin, headache after use, dizziness or nausea). The smartphone app was configured in the participants’ native (Brazilian Portuguese) language to ensure accessibility, with an option to change it to English. After training, participants received the device to use alone according to instructions.

#### Transcranial direct current stimulation

The tDCS was set to deliver direct current to the scalp with C4 and FC6 denoting the cathodal electrodes, and areas C3 and FC5 representing the anodal electrodes (according to 10–20 electroencephalogram system). The tDCS device is equipped with four sponge electrodes, each measuring 2 cm by 6 cm (total surface area of 12 cm^2^) that must be impregnated by the user with a saline (0.9% NaCl) solution before placing it on the head. Each participant self-administered one 20-minute session.

In the active tDCS group, the current was ramped up from 0 to 2.0 mA over 30 seconds, held constant for 19 minutes, and ramped down over 30 seconds, for a total session time of 20 minutes. In the sham tDCS group, in order to ensure blindedness, current was ramped up to 0.5 mA over 30 seconds, then ramped down to 0.01 mA, considered negligible, and held at the negligible output for 19.5 minutes, for a total session duration of 20 minutes [16]. This method produces the same mimic sensations such as itching, and tingling observed during active tDCS [17], enabling blindedness to be achieved. Both active and sham tDCS groups used identical devices, with the session type being determined by a previously randomized code input into the smartphone app. To decrease the risk of incorrect device usage, tDCS devices feature a predetermined (fixed) intensity and duration program (for active or sham groups accordingly). During tDCS use, participants were instructed to feel free and continue with their normal routine.

### Adverse event monitoring and reporting

Adverse events were carefully monitored throughout the clinical usability study in both active and sham conditions, during and after tDCS use [8,17]. Possible adverse events included itching, tingling, burn sensation, headache, nausea and dizziness. After the study, the results were presented to the participants and all participants in the sham group were allowed to test active tDCS.

### Outcomes

In the fourth phase of the study, participants were evaluated using the System Usability Scale (SUS) [18,19] and the Patient Global Impression of Change (PGIC) scale [20]. The SUS questionnaire was translated and validated into Portuguese and consists of 10 questions, 5 of which are positive and 5 are negative [18]. Response options vary on a 5-point scale ranging from strongly disagree [1] to strongly agree [5]. The global score is given on a scale from 0 to 100 points, with 68 being the cut-off point to consider the tool usable [19].

The perception of improvement was assessed according to the PGIC scale. This scale is a simple evaluation method, composed of a 7-item scale, ranging from “1 = no changes” to “7 = much better” in which the patient can broadly assess his/her improvement throughout the treatment, involving the personal and psychometric aspects inherent to the pain treatment process [20]. This scale was adapted and validated for Portuguese and is capable of identifying minimal important clinical changes, sometimes not noticed in pain questionnaires or physical exams [20].

Clinical assessment of each participant was collected solely to characterize the sample. Beck Depression Inventory (BDI) was used to assess depression levels [21]. This is a self-administered questionnaire, which is a self-report instrument composed of 21 items referring to symptoms and cognitive attitudes [21]. For each item, the patient must choose one or more statements that best describe how she has felt in the last week. The maximum score is 63 points, and high scores indicate severe levels of depression [21]. The authors recommend the following quantification points for depression: a score less than 10 indicates no or minimal depression; from 10 to 18 mild to moderate depression; from 19 to 29 moderate to severe depression; from 30 to 63 severe depression [21].

Anxiety was measured using the Hamilton Anxiety Scale (HAS) [22]. This was one of the first rating scales developed to measure the severity of anxiety symptoms and is still widely used today in clinical and research settings [22]. The scale has 14 items, which can be evaluated from 0 to 4, with a total score of 56. The higher the score directed by the individual, the greater the degree of anxiety [22].

Affectivity was assessed using The Positive and Negative Affect Scale (PANAS). This questionnaire consists of a set of 20 words that describe different feelings and emotions felt by the patient during the last 60 weeks [23]. This questionnaire has two dimensions, using 10 words to calculate positive affectivity and 10 for negative affectivity [23]. Each word is scored from 1 (not at all) to 5 (extremely), and the total scores can vary from 10 to 50 for each dimension.

This study also used the Premenstrual Symptoms Screening Tool (PSST) [24]. This questionnaire was developed by Steiner et al. in 2003, translated and validated to Portuguese by Câmara et al. in 2017 [25]. This questionnaire consists of 14 questions related to the presence and intensity of symptoms related to premenstrual syndrome (PMS) and five questions that investigate the interference of these factors in daily activities and relationships of the individual. All items are evaluated using a Likert-type scale, ranging from 0–4, in which 0 = absent, 1 = mild, 2 = moderate and 4 = severe [25]. A positive result for premenstrual syndrome, according to this scale requires: presence of at least five items from the first domain, classified from moderate to severe; at least one of the four main symptoms (anger/irritation, anxiety/tension, desire to cry/increased sensitivity to rejection, depressed mood/hopelessness) classified as moderate or severe; at least one item from the second domain classified as moderate or severe. Participants who do not meet the above requirements are, according to the PSST, classified as having no premenstrual symptoms or having mild symptoms [25].

The World Health Organization Quality of Life Questionnaire short version (WHOQOL-brief) was used to assess quality of life [26]. This questionnaire is composed of 26 questions, where question one and two deal with general quality of life and make up domain five, and the other questions are distributed in four domains: physical domain, psychological domain, social relationships and environmental domain. A higher score indicates better quality of life [26]. This is a helpful tool to assess the impact of PD on participants’ quality of life, social and personal functioning, known to affect overall wellbeing [27].

Lumbar dynamometer (Crown, Brazil) was used to evaluating the individual’s isometric static force. To perform dynamometry each participant being evaluated should stand on the dynamometer platform with their knees fully extended. The trunk will be bent forward forming an angle of approximately 120º. The participant being evaluated should position their head in the extension of the trunk with their gaze fixed forward and their arms extended. The dynamometer cable should be adjusted according to the size of the participant being evaluated, so that they can hold the support bar while maintaining the position described earlier. The support bar should be positioned near the height of the person’s knee. Three tests were performed, and the mean value was used.

The submaximal functional capacity was evaluated through the 6-minute walk test (TC6M) [28]. The 30-second sit-to-stand chair test was performed to measure the strength of the lower limbs. A chair with 43 cm of height and backrest was used, as well as a stopwatch. The score will correspond to the number of times the person can perform the full movement in 30 seconds [28]. The timed up and go (TUG) test was used to evaluate the strength of the lower limbs, power, speed, agility and dynamic balance [28]. The individuals were instructed to stand up from the seated position at the signal of the examiner, walk a comfortable distance of 3 meters, turn, walk back to the chair and sit down again [28].

### Statistical analysis

SPSS software version 19.0 (IBM Corp., Armonk, NY, USA) was used for statistical analyses. Clinical and sociodemographic characteristics were described by means and standard deviations for normal distribution or median, first and third quartiles for non-normally distributed. Categorical outcomes were presented by frequency. A chi-squared test was used to compare the distributions of categorical variables. Shapiro-Wilk test was used to assess the normality of the distribution. To compare data between groups, an unpaired t-test or Mann-Whitney were used. Statistical significance was set at p < 0.05.

## Results

A total of forty-nine women completed all procedures of the clinical usability study. There was no dropout during the study protocol. No participants were familiar with the tDCS protocol nor had previous experiences with non-invasive brain stimulation. No critical clinical problems related to assessment, tDCS and smartphone app were observed during the research. All participants tolerated the tDCS well with minimum side effects reported of itching and tingling sensations.

Table 1 showed the sociodemographic and clinical characteristics for both groups. No clinical difference was found. Both groups have women of reproductive age and classified as young adults. No difference between groups was found for body mass index, muscle strength, submaximal aerobic capacity, agility, speed, quality of life and premenstrual symptoms.

**Table 1:** Sociodemographic and clinical characteristics.

Variable	Active Group (n = 24)	Sham Group (n = 25)	*P* value
Age (years)	22.5 (21–25.7)	22 (20–23)	0.17
Age of menarche (years)	12 (11–14)	12 (11–13.5)	0.82
BMI (Kg/cm^2^)	23.1 (21.4–25.3)	22.9 (21.1–26)	0.42
Dynamometry (kgf/cm^2^)	69.3 (58.7–75.7)	72.6 (59–81.6)	
Sit and stand up	18 (14–24.5)	17 (14–23.5)	0.45
6MWT	500 (476.3–544.4)	480 (455–527.8)	0.29
TUG	7.46 ± 1.18	7.76 ± 0.85	0.30^†^
WHOQOL			
Physical Domain	3.22 ± 0.41	3.18 ± 0.32	0.72^†^
Psychology Domain	3.5 ± 0.4	3.5 ± 0.41	0.96^†^
Social Relation Domain	3.83 (3.67–4.58)	4 (3.67–4.33)	0.50
Environment Domain	3.83 ± 0.48	3.72 ± 0.63	0.48^†^
BDI	7.5 (5–14.5)	9 (4.5–15)	0.82
PSST (n/%)			0.89
Positive	13 (54.16)	14 (56)	
Negative	11 (45.83)	11 (44)	
Income (%)			0.41
Up to 1 MW	3 (12.5)	5 (20)	
2–3 MW	10 (41.7)	7 (28)	
More than 4 MW	10 (41.7)	9 (36)	
Not respond	1 (4.2)	4 (16)	
Education (%)			0.05
Elementary	0 (0.00)	0 (0.00)	
Secondary	1 (4.16)	0 (0.00)	
College	17 (70.8)	24 (100)	
University	6 (25)	1	
Marital Status (%)			0.47
Single	18 (75)	25 (100)	
Married	5 (20.8)	0 (0.00)	
Divorced	1 (4.2)	0 (0.00)	
Race (%)			0.44
White	14 (58.3)	14 (56)	
Asian or Pacific Islander	2 (8.3)	0 (0.00)	
Mixed	5 (20.8)	8 (32)	
Black	3 (12.5)	3 (12)	
Hair (%)			0.92
traight	10 (47.7)	9 (36)	
Wavy	7 (29.2)	7 (28)	
Curly	7 (29.2)	9 (36)	

BMI—body mass index. MW—minimum wage. 6MWT—six-minute walk test. TUG—Time up and go. WHOQOL—World Health Organization Quality of Life. HAM-A—Hamilton Anxiety Rating Scale. Beck’s Depression Inventory. PSST—Premenstrual symptoms screening tool.

*Significance for p < 0.05.

^†^Unpaired t-tests used. It used the means and standard deviations for normally distributed data and medians, first and third quartiles for non-normally distributed data.

**Fig 3:**
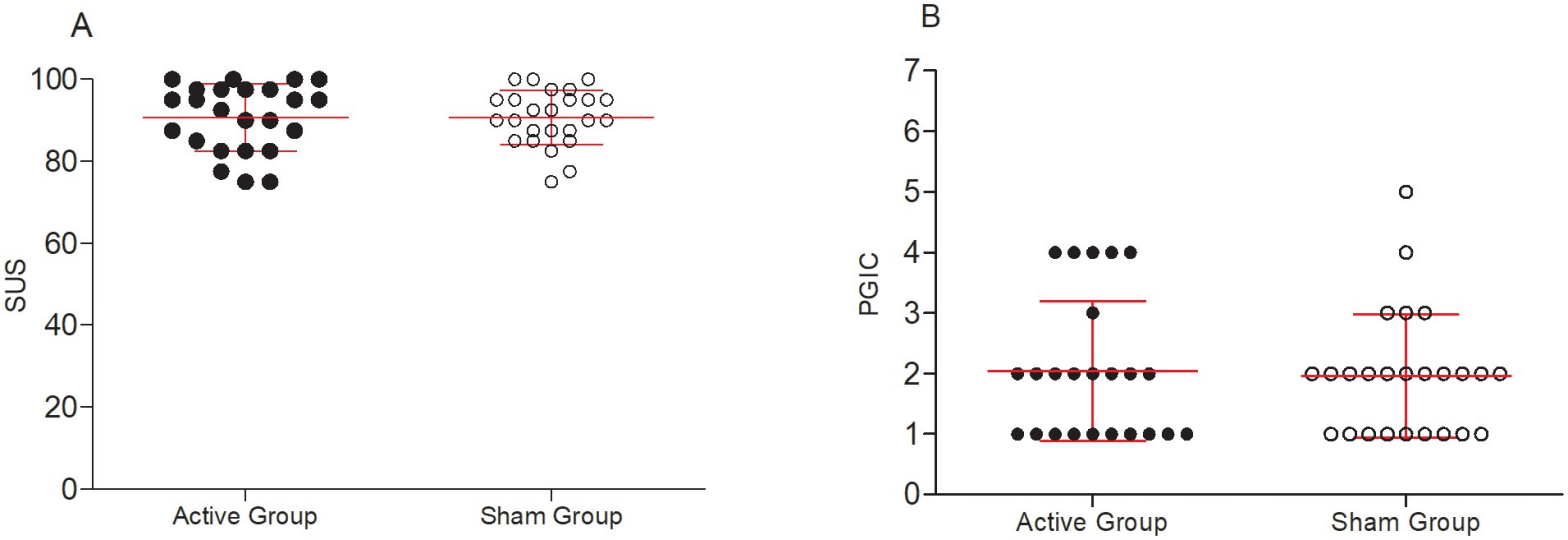
(A) Score of the System Usability Scale (SUS). (B) Patient Global Impression of Change (PGIC) for active and sham groups. No significant difference was found between groups.

No significant difference was found between groups for SUS (Active group: 93.7 (83.1–97.5); Sham group 90 (86.2–95) p = 0.79) and PGIC (Active group: 2 (1–2.75); Sham group 2 (1–2) p = 0.99) (Fig 3). No difference was found for each item of SUS (Table 2). This follows expectations, as the system should be used equivalently by both active and sham groups in order to be appropriately, clinically tested in a sham-controlled clinical setting. The low PGIC scores are expected given that the sample studied for usability purposes was not in the menstrual period.

**Table 2:** System usability index results.

Questions	Active Group (n = 24)	Sham Group (n = 25)	*p* value
Likely frequently use the system	4 (3–4)	4 (3–5)	0.58
Unnecessarily complicated	1 (1–1)	1 (1–1)	0.74
Easy usage	5 (5–5)	1 (1–1.5)	0.62
Necessary help in using	1 (1–1)	5 (4–4)	0.84
Well-integrated functions	5 (4.25–5)	4 (3–4)	0.69
Presence of inconsistencies	1 (1–1)	1 (1–1)	0.50
Imagine that people will easily learn how to use	5 (4–5)	5 (5–5)	0.46
Clumsy to use	1 (1–1)	1 (1–1)	0.98
A feeling of confidence during the usage	5 (4–5)	5 (4–5)	0.71
Need to learn new information to use	1 (1–1)	1 (1–2)	0.37
Total	93.7 (83.1–97.5)	90 (86.2–95)	0.79

Mann-Whitney test was used. Values in medians, first and third quartiles.

**Fig 4:**
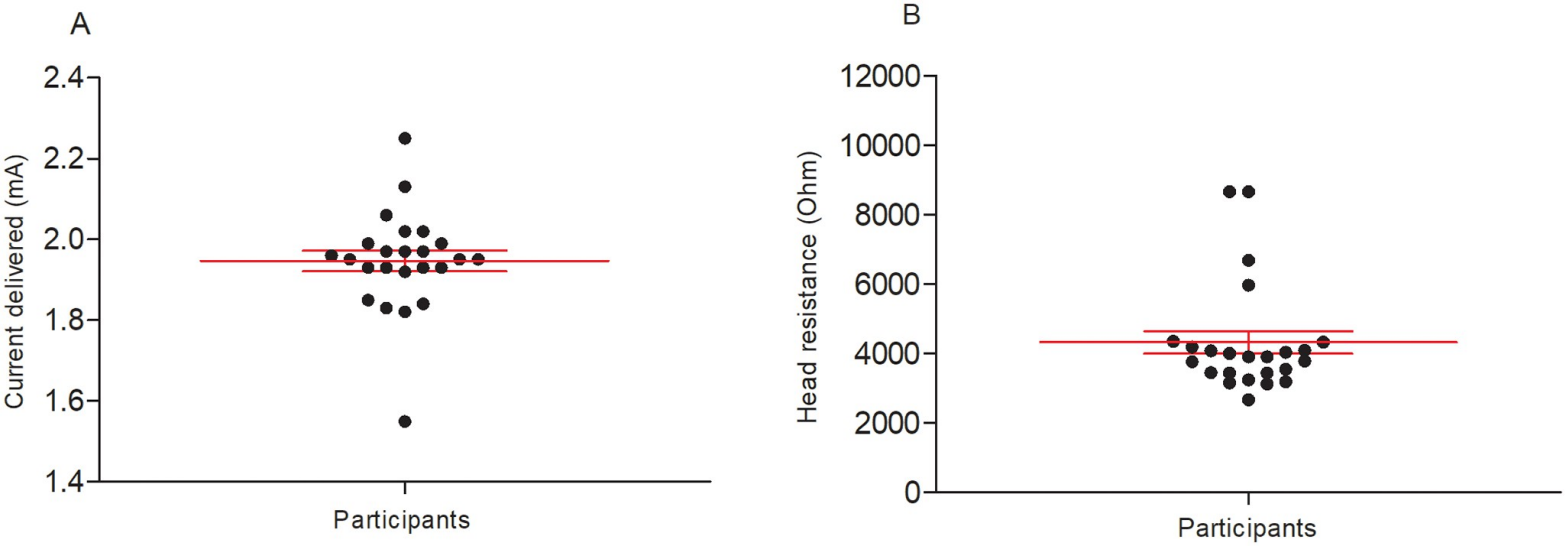
Effectiveness of current delivery to the active group. Longer lines indicate median current and head resistance, and whiskers indicate the first and third quartiles.

To confirm that those who received active stimulation parameters (2.0 mA) actually had current delivered, we measured individually delivered current and head resistance, which is influenced by skin, skull and hair variables, among others. As expected, in the active group the mean delivered current was 2.03 mA and the mean head resistance was 4.1 kOhms, which are both in the expected effective range (Fig 4).

## Discussion

The tDCS device and its respective mobile application were tested through a clinical usability sham-controlled study. Forty-nine women completed all phases of the study. The active group exhibited a median usability evaluation (SUS) score of 9.37, signifying an exceptional level of usability and satisfaction for the product. This result is notably higher than the industry benchmark, which typically accepts devices with a mean SUS score of 68 for usability purposes [18,19].

SUS scores evaluate self-reported data collection about the characteristics, thoughts, feelings, perceptions, behaviors or attitudes about a product [18]. It is essential that new home-based tDCS is easy to use and accepted by patients for long-time use. Protocols with safety guidance are needed to avoid the incorrect use of the device and guarantee that correct parameters are employed. To reduce this risk, most home-based remotely supervised tDCS devices have a fixed intensity and duration program, while others can be remotely controlled and adjusted by a specialized technician [12,29,30]. PGIC is used to analyze the perception of improvement after an intervention [20]. Given that this is a usability study rather than an evaluation of treatment effectiveness, the selected population does not exhibit notable symptoms of pain or premenstrual dysphoric syndrome during the tDCS sessions and subsequent assessments. Additionally, it’s essential to note that only a single tDCS session was administered, which constitutes a relatively low dosage to anticipate any significant clinical improvement [32]. If the study population had comprised individuals with higher pain scores, it would likely have facilitated the discernment of distinctions between the active and sham groups concerning the PGIC.

The tDCS device used in this study incorporates remote IoT technology and is operated by a mobile app. It can be used at home, outside the hospital or clinical environment during routine activities. This device was developed with a focus on women’s health, aiming at improving menstrual pain and mood challenges. In the process, over a hundred women contributed to design, comfort and ergonomic choices to ensure that the device fulfills not only a functional, but also an aesthetic and comfort function to increase its adoption among users. Ensuring safety during home-based self-administration of tDCS necessitates the establishment of predetermined parameters [17]. This is essential to ensure proper current intensity usage and acceptable resistance levels, thereby mitigating potential adverse effects such as pronounced itching, tingling, and burning sensations [17]. Throughout the study protocol, the current delivery and head resistance remained within the expected effective range.

Previous studies have shown that the use of anodic tDCS over dorsolateral prefrontal cortex (DLPFC) or M1 improves pain and anxiety symptoms in women with primary dysmenorrhea [31,32]. Both areas are involved in the pain modulatory system and mood disturbance [9,10,33]. The activation of the DLPFC and M1 is associated with deep brain modulation and the regulation of pain intensity, encompassing affective and cognitive aspects [7]. Electroencephalography studies suggest a decreased activity of the left DLPFC in depression and the anode placed over F3 could facilitate the activity of this brain area [34]. Furthermore, the DLPFC is associated with anxiety regulation and emotional aspects of pain according to the modulatory mechanisms of limbic reactivity [35]. Pain, depression and anxiety are strongly associated in some women’s health issues including primary and secondary dysmenorrhea, pelvic pain, interstitial cystitis and endometriosis [1,4,36].

For mood disorders, home-based remotely supervised tDCS offers an alternative treatment, with an advantage for portability, ease of use and absence of severe adverse effects. Mood disorders are a common state in women’s lives, especially during menstrual periods, gestation or in menopause. The potential application of tDCS in conjunction with remote IoT technology for women’s health represents a significant and innovative prospect that is yet to be tested in future clinical trials focused on efficacy.

## Limitations

This study is the first in a line of experiments aiming to test this wearable home-based self-administration tDCS system for its application in women’s health. It therefore does not address the use of specific protocols, or does not discuss the expected clinical effects, of such treatment. However, it validates not only the need for such innovation, but also the ability of this tested system to be used for future testing applications.

## Conclusion

The home-based self-administration tDCS and the app showed excellent usability and satisfaction by users. The use of a portable tDCS device, characterized by its user-friendly interface and the option for in-home stimulation, has the potential to enhance patient adherence to treatment. This approach offers a novel avenue for addressing pain and mood-related concerns associated with women’s menstrual health that is yet to be tested.

## Supporting information

S1 Checklist CONSORT 2010 checklist of information to include when reporting a randomised trial*. (DOC)

S1 File (PDF)
