# Supplementary material for: Clinical usability study of a home-based self-administration transcranial direct current stimulation for primary dysmenorrhea: A randomized controlled trial
Source: PLoS One. 2024 May 2;19(5):e0301851. doi: 10.1371/journal.pone.0301851 (PMC11065276; doi:10.1371/journal.pone.0301851)
Supplement: S1 File — (PDF) [file pone.0301851.s002.pdf]

Brazilian Registry of Clinical Trials (ReBEC)

RBR-56w7h2p Electrical Stimulation in women with Primary Dysmenorrhea

Date of registration: 05/17/2023 **Last approval date :** 05/17/2023

Study type:
Interventional

Scientific title:

en
Transcranial Direct Current Stimulation in women with Primary Dysmenorrhea: usability and clinical trial

pt-br
Estimulação Transcraniana por Corrente Contínua em mulheres com Dismenorreia Primária: usabilidade e ensaio clínico

es
Transcranial Direct Current Stimulation in women with Primary Dysmenorrhea: usability and clinical trial

Trial identification

UTN code: U1111-1282-0975

Public title:

en
Electrical Stimulation in women with Primary Dysmenorrhea

pt-br
Estimulação Elétrica em mulheres com Dismenorreia Primária

Scientific acronym:

Public acronym:

Secondaries identifiers: **58806322.3.0000.5537** Issuing authority: Plataforma Brasil
5.792.825 Issuing authority: Comitê de Ética em Pesquisa da Universidade Federal do Rio Grande do Norte

Sponsors

Primary sponsor: Universidade Federal do Rio Grande do Norte
Secondary sponsor: **Institution:** Universidade Federal do Rio Grande do Norte

Supporting source: **Institution:** Universidade Federal do Rio Grande do Norte

Health conditions

en
Primary dysmenorrhea

pt-br
Dismenorreia primária

General descriptors for health conditions:

en
C23.550.568 Menstruation Disturbances

pt-br
C23.550.568 Distúrbios Menstruais

Specific descriptors:

en
N944 Primary dysmenorrhea

pt-br
N944 Dismenorreia primária

Interventions

Interventions:

en
This is a randomized, controlled, parallel, double-blind, single-center clinical trial to evaluate the effects of tDCS in women with primary dysmenorrhea. Experimental group: 20 women with primary dysmenorrhea will receive 5 sessions of transcranial electrical stimulation. Sham group: 20 women with primary dysmenorrhea will receive 5 sessions of sham-type transcranial electrical stimulation. Sessions will be held on consecutive days with one session per day. Participants will be randomly allocated into two groups: active group and sham group. Randomization will be performed through a numerical sequence generated by a computer using appropriate software (www.random.org) to assign each participant to the active or sham group. An external survey assistant will generate the allocation sequence and contact participants by phone. Allocation concealment will be performed using opaque envelopes. Participants and researchers involved in assessments and interventions will be blinded to group allocation throughout the study. The study will be double blind, where the evaluator will not know which group each participant belongs to and the participants will not know what type of therapy they will undergo (sham or active). The evaluator will be a trained research physiotherapist and will not be present at any other stage of the study.

pt-br

Trata-se de um estudo do tipo ensaio clínico randomizado e controlado, paralelo, duplo cego, de centro único para avaliar os efeitos da ETCC em mulheres com dismenorreia primária. Grupo experimental: 20 mulheres com dismenorreia primária receberão 5 sessões de estimulação elétrica transcraniana. Grupo sham: 20 mulheres com dismenorreia primária receberão 5 sessões de estimulação elétrica transcraniana do tipo sham. As sessões serão realizadas em dias consecutivos com uma sessão por dia. Os participantes serão alocados aleatoriamente em dois grupos: grupo ativo e grupo sham. A randomização será realizada por meio de uma sequência numérica gerada por um computador usando um software apropriado (www.random.org) para atribuir cada participante ao grupo ativo ou sham. Um assistente de pesquisa externo gerará a sequência de alocação e contatará os participantes por telefone. A ocultação de alocação será realizada usando envelopes opacos. Os participantes e pesquisadores envolvidos nas avaliações e intervenções serão cegos para alocação de grupo durante todo o estudo. O estudo será duplo cego, onde o avaliador não saberá que grupo pertence cada participante e as participante não saberão que tipo de terapia realizará (sham ou ativa). O avaliador será um fisioterapeuta pesquisador capacitado e não estará presente em nenhuma outra etapa do estudo.

Descriptors:

en

E02.331.750 Transcranial Direct Current Stimulation

pt-br

E02.331.750 Estimulação Transcraniana por Corrente Contínua

Recruitment

Study status: Recruiting
Countries Brazil

Date first enrollment: 04/01/2023

Target sample size:	Gender:	Minimum age:	Maximum age:
40	F	18 Y	45 Y

en

Age between 18 and 45 years; medical diagnosis of primary dysmenorrhea according to Guideline No. 34539; mean pain equal to or greater than 4 on the Visual Analogue Scale of Pain; periodic pain of at least 3 menstrual cycles in a row; having a regular menstrual cycle of 28 to 32 days; no pelvic inflammatory disease; history of head trauma, epilepsy, endometriosis, current pregnancy, chronic abdominal pain unrelated to the menstrual cycle; fibroids; inflammatory bowel syndrome; irritable bowel syndrome; major abdominal or pelvic surgery; alcohol dependence; nicotine or drugs,; metallic implant; pacemaker; and chronic urinary tract disease

pt-br

Idade entre 18 a 45 anos; diagnóstico médico de dismenorreia primária de acordo com a Diretriz Nº 34539; média de dor igual ou maior a 4 na Escala Visual Analógica da Dor; dor periódica de pelo menos 3 ciclos menstruais seguidos; possuir ciclo menstrual regular de 28 a 32 dias; não apresentar doenças inflamatórias pélvicas, história de traumatismo craniano, epilepsia, endometriose, gravidez vigente, dor abdominal crônica não relacionada ao ciclo menstrual; miomas; síndrome do intestino

inflamatório; síndrome do intestino irritável; grande cirurgia abdominal ou pélvica; dependência de álcool; nicotina ou drogas; implante metálico; marcapasso; e doença crônica do trato urinário

Exclusion criteria:

en

Severe headache in more than two transcranial stimulation sessions; dizziness or severe migraine in more than two transcranial stimulation sessions; failure to perform 2 or more transcranial stimulation applications

pt-br

Dor de cabeça intensa em mais de duas sessões de estimulação transcraniana; tontura ou enxaqueca forte em mais de duas sessões de estimulação transcraniana; não realização de 2 ou mais aplicações de estimulação transcraniana

Study type

Study design:

Expanded access program	Purpose	Intervention assignment	Number of arms	Masking type	Allocation	Study phase
1	Treatment	Parallel	2	Double-blind	Randomized-controlled	N/A

Outcomes

Primary outcomes:

en

It is expected to find a moderate improvement of 30% to 50% in pain determined through the visual analogue scale. Data collected one week before the intervention, at the end of the intervention and with a 30-day follow-up

pt-br

Espera-se encontrar uma melhora moderada de 30% a 50% na dor determinada através da escala visual analógica. Dados coletados uma semana antes da intervenção, ao final da intervenção e com um follow-up de 30 dias

Secondary outcomes:

en

Expected to find moderate improvement in pressure pain threshold and tolerance using a pressure algometer

pt-br
Espera-se encontrar melhora moderada no limiar e tolerância à dor à pressão utilizando um algômetro de pressão

en
It is expected to find a moderate improvement of 30% to 50% in the intensity and interference of pain in the lives of volunteers through the pain inventory. Data collected one week before the intervention, at the end of the intervention and with a 30-day follow-up

pt-br
Espera-se encontrar uma melhora moderada de 30% a 50% na intensidade e na interferência da dor na vida dos voluntários através do inventário de dor. Dados coletados uma semana antes da intervenção, ao final da intervenção e com um follow-up de 30 dias

en
It is expected a slight improvement in the submaximal functional capacity assessed through the 6-minute walk test is expected. Data collected one week before the intervention, at the end of the intervention and with a 30-day follow-up

pt-br
Espera-se melhora leve da capacidade funcional submáxima avaliado através do teste de caminhada de 6 minutos. Dados coletados uma semana antes da intervenção, ao final da intervenção e com um follow-up de 30 dias

en
It is expected a slight improvement of up to 20% is expected in lower limb strength according to the 30-second sit-to-stand test. Data collected one week before the intervention, at the end of the intervention and with a 30-day follow-up

pt-br
Espera-se melhora leve de até 20% na força dos membros inferiores de acordo com o teste de sentar e levantar da cadeira em 30 segundos. Dados coletados uma semana antes da intervenção, ao final da intervenção e com um follow-up de 30 dias

en
It is expected a slight improvement of up to 20% in power, speed, agility and dynamic balance is expected according to the Timed Up and Go test. Data collected one week before the intervention, at the end of the intervention and with a 30-day follow-up

pt-br
Espera-se melhora leve de até 20% na potência, velocidade, agilidade e equilíbrio dinâmico de acordo com o teste Timed Up and Go. Dados coletados uma semana antes da intervenção, ao final da intervenção e com um follow-up de 30 dia

en
It is expected a moderate improvement of up to 30% is expected in quality of life assessed by the short version of the World Health Organization Quality of Life questionnaire. Data collected one week before the intervention, at the end of the intervention and with a 30-day follow-up

pt-br
Espera-se melhora moderada de até 30% na qualidade de vida avaliada pelo questionário de Qualidade de Vida da Organização Mundial da Saúde versão breve. Dados coletados uma semana antes da intervenção, ao final da intervenção e com um follow-up de 30 dias

en
A moderate improvement of up to 30% is expected in positive and negative affect assessed by the affect scale. Data collected one week before the intervention, at the end of the intervention and with a 30-day follow-up

pt-br
Espera-se melhora moderada de até 30% no afeto positivo e negativo avaliado pela escala de afeto. Dados coletados uma semana antes da intervenção, ao final da intervenção e com um follow-up de 30 dias

en
A moderate improvement of up to 30% is expected in anxiety measured according to the Hamilton Anxiety Scale. Data collected one week before the intervention, at the end of the intervention and with a 30-day follow-up

pt-br
Espera-se melhora moderada de até 30% na ansiedade medido de acordo com a escala de ansiedade de Hamilton. Dados coletados uma semana antes da intervenção, ao final da intervenção e com um follow-up de 30 dias

en
A moderate improvement of up to 30% is expected in depression measured according to the Beck Depression Scale. Data collected one week before the intervention, at the end of the intervention and with a 30-day follow-up

pt-br
Espera-se melhora moderada de até 30% na depressão medido de acordo com a escala de depressão de Beck. Dados coletados uma semana antes da intervenção, ao final da intervenção e com um follow-up de 30 dias

en
High improvement in symptoms related to primary dysmenorrhea as assessed by the Premenstrual Symptom Screening Tool and the Somatic Symptom Scale-8. Data collected one week before the intervention, at the end of the intervention and with a 30-day follow-up

pt-br

Melhora alta nos sintomas relacionados a dismenorreia primária avaliado pela Ferramenta de Triagem de Sintomas Pré-Menstruais e e Escala de Sintomas Somáticos-8. Dados coletados uma semana antes da intervenção, ao final da intervenção e com um follow-up de 30 dias
